# Ion Channel Clustering at the Axon Initial Segment and Node of Ranvier Evolved Sequentially in Early Chordates

**DOI:** 10.1371/journal.pgen.1000317

**Published:** 2008-12-26

**Authors:** Alexis S. Hill, Atsuo Nishino, Koichi Nakajo, Giuxin Zhang, Jaime R. Fineman, Michael E. Selzer, Yasushi Okamura, Edward C. Cooper

**Affiliations:** 1 Penn Epilepsy Center, Department of Neurology, University of Pennsylvania, Philadelphia, Pennsylvania, United States of America; 2 Mahoney Institute of Neurological Sciences, Philadelphia, Pennsylvania, United States of America; 3 Laboratory of Developmental Biology, Department of Biology, Graduate School of Science, Osaka University, Osaka, Japan; 4 National Institute for Physiological Sciences, Okazaki, Japan; 5 Office of Research and Development, US Department of Veterans Affairs, Washington, D.C., United States of America; 6 Department of Integrative Physiology, Graduate School of Medicine, Osaka University, Osaka, Japan; The Jackson Laboratory, United States of America

## Abstract

In many mammalian neurons, dense clusters of ion channels at the axonal initial segment and nodes of Ranvier underlie action potential generation and rapid conduction. Axonal clustering of mammalian voltage-gated sodium and KCNQ (Kv7) potassium channels is based on linkage to the actin–spectrin cytoskeleton, which is mediated by the adaptor protein ankyrin-G. We identified key steps in the evolution of this axonal channel clustering. The anchor motif for sodium channel clustering evolved early in the chordate lineage before the divergence of the wormlike cephalochordate, amphioxus. Axons of the lamprey, a very primitive vertebrate, exhibited some invertebrate features (lack of myelin, use of giant diameter to hasten conduction), but possessed narrow initial segments bearing sodium channel clusters like in more recently evolved vertebrates. The KCNQ potassium channel anchor motif evolved after the divergence of lampreys from other vertebrates, in a common ancestor of shark and humans. Thus, clustering of voltage-gated sodium channels was a pivotal early innovation of the chordates. Sodium channel clusters at the axon initial segment serving the generation of action potentials evolved long before the node of Ranvier. KCNQ channels acquired anchors allowing their integration into pre-existing sodium channel complexes at about the same time that ancient vertebrates acquired myelin, saltatory conduction, and hinged jaws. The early chordate refinements in action potential mechanisms we have elucidated appear essential to the complex neural signaling, active behavior, and evolutionary success of vertebrates.

## Author Summary

Because nervous systems generate behavior, innovations that confer new neuronal signaling functions are important potential factors in evolution. In mammals, clustering of ion channels on nerves is essential for electrical impulses used in rapid signaling. This channel clustering is generally absent in insects, worms, and other non-chordates. We traced the evolutionary emergence of mechanisms underlying channel clustering on nerves by analyzing the genomes of primitive chordates and studying the cellular distribution and functional properties of their channels. We found that sodium channel clustering evolved early in the chordate lineage, before the divergence of the earliest wormlike and planktonic groups (lancelets and sea squirts). Nerve fibers of the lamprey, a primitive fish, retained some invertebrate features but possessed dense sodium channel clusters like in more recently evolved vertebrates. A potassium channel clustering system evolved, after the divergence of lampreys, in a common ancestor of shark and humans. We conclude that the clustering of sodium channels on axons was the initial pivotal step in a chordate-specific series of evolutionary innovations, making nerve impulses more rapid and robust. The refinements in action potentials we have elucidated appear essential for the complex neural signaling and active behavior of vertebrates.

## Introduction

**Figure 1:**
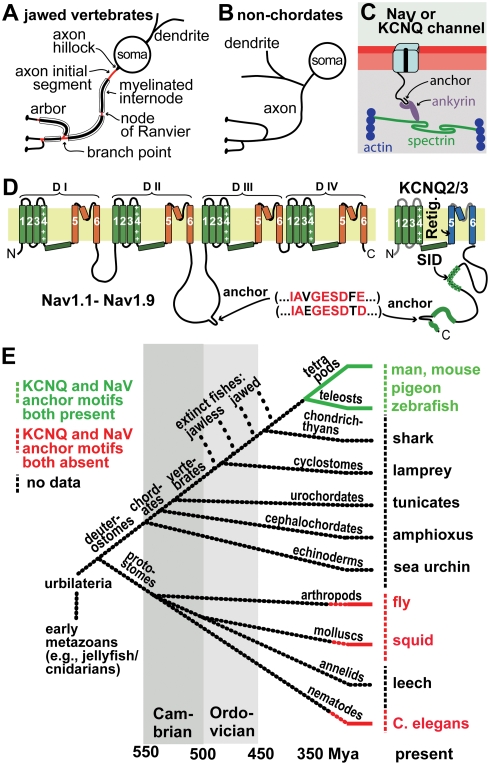
Axonal ankyrin-dependent Na_V_ and KCNQ2/3 channel clusters and anchor motifs: neuronal cellular and molecular features associated with jawed vertebrates and absent from non-chordates invertebrates. (A, B) Cartoons showing characteristic jawed vertebrate (A) and non-chordate (B) types of neuronal polarity. Many jawed vertebrate neurons have myelinated axons, and axonal domains bearing ankyrin-dependent channel clusters, which mediate AP initiation and conduction (AISs, nodes, and branch points, red). Non-chordate dendrites and axons arise from a common neurite, and lack myelin and channel clusters. (C) Proposed molecular interactions between jawed vertebrate axonal Na_V_ and KCNQ channels, ankyrin-G, spectrin, and actin. (D) Cartoons showing Na_V_ and KCNQ2/3 channel topology. Locations of peptide sequences required for KCNQ opener interaction (Retig., retigabine), tetramerization (SID, subunit interaction domain), and the axonal anchor motif are indicated. (E) Cladogram showing some nomenclature and important evolutionary relationships among animals; timeline is approximate. At right are listed model species whose channel sequences were previously shown [20] to lack anchor motifs (red) or bear them (green), and those newly studied here (black).

Most animals, from jellyfish to man, rely on electrical impulses called action potentials (APs) for rapid, long-distance neuronal signaling. Although APs are nearly always based on flows of sodium and potassium ion currents through voltage-gated channel proteins [1], comparisons across phyla reveal important differences in the ways that APs are initiated and conducted [2]–[4]. In jawed vertebrates (i.e., sharks, jawed bony fish, and tetrapods), the rate of AP propagation along nerve fibers, or axons, is markedly increased by myelin, an insulating coating around the axon formed by glia, and by nodes of Ranvier, small gaps in the myelin where dense clusters of ion channels boost the AP signal. Most vertebrate neurons also possess a robust and stereotyped polarity of form and function, with well-segregated domains for reception and integration of synaptic inputs (the dendrites, soma and proximal axon), AP initiation (the proximal axon) and rapid propagation (the axonal arbor) (Figure 1A). By contrast, invertebrate neurons typically lack myelinated axons, and their afferent and efferent processes often branch from a common offshoot of the soma (Figure 1B). These typical morphological differences between vertebrate and invertebrate neurons were well appreciated by the early anatomist Ramon y Cajal [5]. More recently, physiological studies of invertebrate axons have revealed functional properties uncharacteristic of vertebrates, such as proximal axons that lack the ability to initiate APs, spikes whose initiation and propagation are confined to particular axon branches, and initiation locations that vary dynamically, depending on the sites and temporal pattern of synaptic inputs [6]–[10]. The biophysical and molecular reasons underlying apparent differences in AP initiation between vertebrates and invertebrates have been poorly understood.

In mammals, similar membrane-associated protein complexes mediate AP initiation by the proximal axon and AP conduction by nodes of Ranvier [11]–[14]. The axon hillock has no special role in AP initiation. Instead, at both the “axon initial segment” (AIS), a 10–60 µm long axonal unmyelinated domain bounded by the hillock and the first internode, and at the nodes, voltage-gated sodium (Na_V_) channels are concentrated at high densities, generating large transient inward currents that rapidly depolarize the membrane potential. Na_V_ channel concentration at the AIS and node both depend upon a specialized membrane cytoskeleton of actin-spectrin modules [12], [15]–[18]. The actin-spectrin network is linked via the adaptor, ankyrin-G, to Na_V_ channels, neurofascin 186 (a L1 family cell adhesion molecule), and the voltage-gated potassium ion (K_V_) channel subunits, KCNQ2 and KCNQ3 (Figure 1C) [19]–[21]. KCNQ2 and KCNQ3 (also called Kv7.2 and Kv7.3) mediate an extensively studied neuronal current (M-current or I_M_), which dampens and modulates excitability in many neurons [22],[23]. Indeed, genetic and electrophysiological studies indicate that KCNQ channels at AISs and nodes of Ranvier strongly modulate excitability [24]–[27]. Mutations that diminish the clustering of Na_V_ and KCNQ channels at AISs lead to recurrent epileptic seizures [28],[29]. The medical importance of better understanding of axonal Na_V_ and KCNQ channels is further underlined by the fact that these channels are targets of many drugs approved and in development for epilepsy, psychiatric, and pain syndromes [30]–[32].

A model of the molecular mechanisms by which ankyrin-G clusters mammalian Na_V_, KCNQ2, and KCNQ3 channels at the AIS and node has emerged from studies of nerve and muscle cells in vitro and in transgenic mice, and by analogy with better understood protein interactions between ankyrin-G homologues and their binding partners. Na_V_, KCNQ2, and KCNQ3 polypeptides all possess cytoplasmic anchor motifs that share the sequence IAxGESDxD/E and are required for their immobilization at the AIS (Figure 1C–D) [17],[18],[20]. Ankyrin-G, like its homologues ankyrin-R (erythrocytes) and ankyrin-B (expressed widely), possesses a membrane interaction domain consisting of 24 solenoidal ankyrin repeats. Mutagenesis experiments indicate that ankyrin-G repeats 13–15 mediate interaction with the Na_V_ channel anchor (Figure 1C) [17],[18],[33]. Although the structural basis for ankyrin-G/channel interaction is unknown, studies of ankyrin interactions with cytoplasmic domains of the Na/K-ATPase and erythrocyte band III proteins indicate that adjoining ankyrin repeats form sites for binding short loops protruding from membrane protein cytoplasmic domains [34],[35]. Available cell biological data suggests a similar mode of interaction between ankyrin-G and the Na_V_ and KCNQ2/3 anchor sequences [13],[17],[18],[20],[21],[28].

Although colocalization of channels *per se* is not uncommon, initial studies raised a series of questions about how mammalian Na_V_, KCNQ2 and KCNQ3 channels had evolved such similar ankyrin interaction sequences [20]. BLAST search identified no other mammalian proteins bearing the anchor motifs. A first phylogenetic survey revealed that the Na_V_ and KCNQ anchor motifs were extremely well conserved through over 350 million years of vertebrate evolution, from teleost fish to man, but were absent from the homologous channels of fly, squid and worm (Figure 1E) [20]. Na_V_ and K_V_ channels (including the five members of the KCNQ subfamily, KCNQ1-5) share a common ancestor gene, but these channel families diverged very early, possibly in prokaryotes [1]. How did ancestors of vertebrates, subsequent to their divergence from insects, mollusks, and nematodes, evolve such similar sequences playing similar functions in two unrelated gene families? What was the biological significance of this apparent molecular convergence [36]? Why do all mammalian Na_V_ channels possess anchor motifs, but only KCNQ2 and KCNQ3 among the five KCNQ subunits?

Here, using molecular phylogenetic analysis, we have reconstructed a sequence of evolutionary events through which mammalian Na_V_ and KCNQ channels acquired their anchor motifs. Fly and worm, the model invertebrates most frequently studied by molecular neurobiologists, are protostomes, separated from vertebrates by an important evolutionary gap (Figure 1E). This gap encompasses the Cambrian explosion and its initial aftermath, when the extant bilaterian phyla and subphylum vertebrata suddenly emerged [37],[38]. By obtaining and analyzing sequences from newly available basal deuterostome genomes, we infer how new channel genes and functions arose in early chordates during the Cambrian and Ordovician Periods (∼550–450 Mya, Figure 1E). We show that the Na_V_ channel anchor mechanism first appeared early in this interval, in an invertebrate deuterostome ancestral to all extant chordates. The KCNQ channel anchor first appeared at the very end of this period, in the interval between the divergence of extant jawless and jawed fish (lampreys and sharks). Lamprey axons lack myelin, but those of sharks possess it [2],[39]. Thus, KCNQ anchors appeared during the evolutionary interval when many other proteins evolved mechanisms incorporating them into the axo-glial apparatus of saltatory AP conduction. These findings reveal the stepwise origins in basal chordates of a distinctive vertebrate mechanism underlying excitability and polarity. They show that the node of Ranvier is a secondarily evolved feature, based upon the much earlier evolution of Na_V_ channel clustering mechanisms in invertebrate chordates. We suggest (see Discussion) that these Na_V_ channel clusters be termed excitozones.

## Results

### The Sodium Channel Anchor Motif Is a Shared Exclusive Feature of Chordates

**Figure 2:**
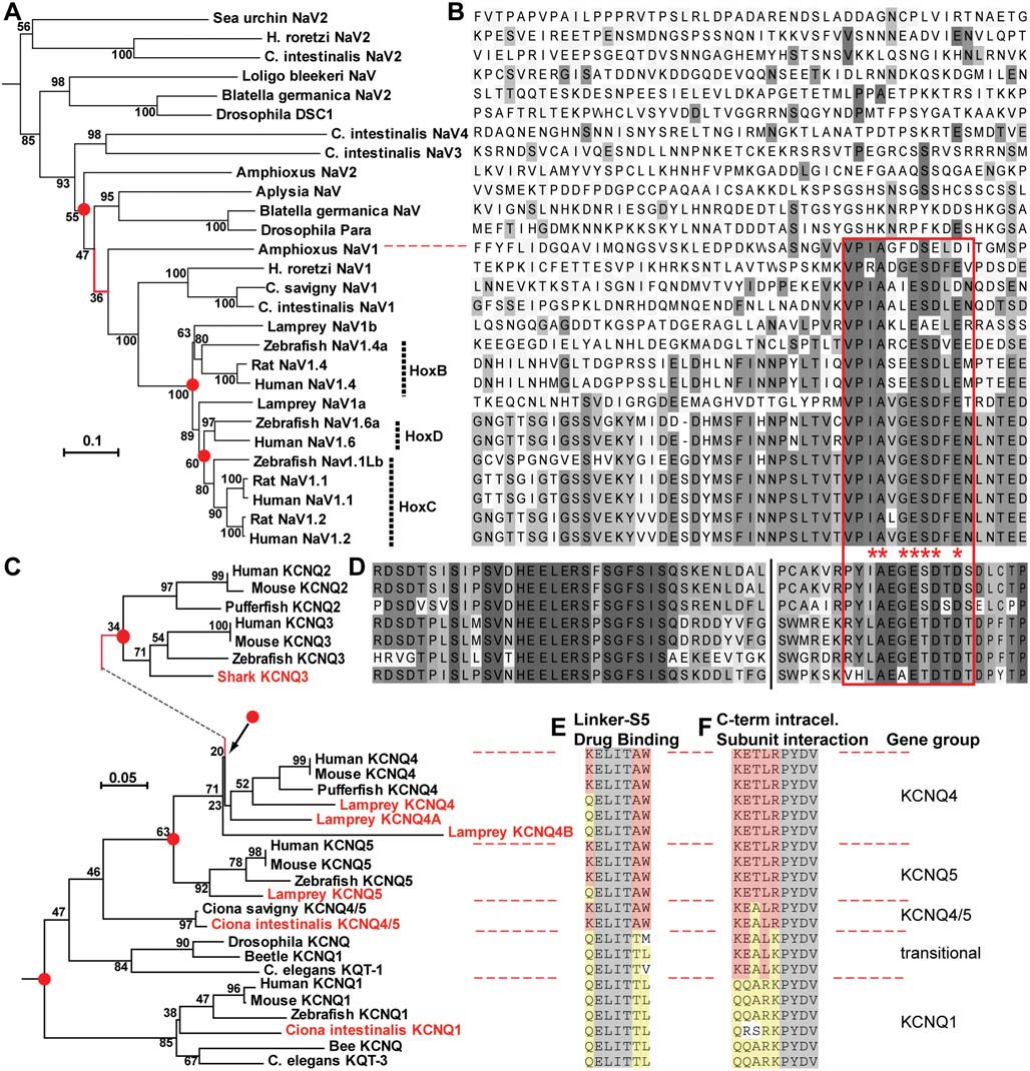
Phylogenetic analysis reveals that anchor motifs evolved sequentially in chordates (Na_V_ channel) and jawed vertebrates (KCNQ2/3). (A) Phylogram (minimal evolution) of Na_V_ channels, showing that all vertebrate channels are derived from chordate NaV1. The branch on the phylogram in which the anchor motif first evolved is shown in red. Key nodes, associated with gene duplications, have red dots. Nodes are labeled with bootstrap values. (B) Alignment of Na_V_ channel DII–DIII loop sequences, showing presence of anchor motifs in chordate NaV1 and all vertebrate channels (below dotted red line). The anchor motifs are boxed (red). Shading indicates each residue's conservation within the aligned 28 Na_V_ sequences: bins represent ≤10, 11–20, 21–30, 31–45, 46–60, and 61–100% conservation. (C) Phylogeny of KCNQ channels, based on analysis of amino acids encoded on exons 5–7. Novel genes identified or cloned in this study are highlighted (named in red) As in A, key nodes associated with gene duplications are highlighted with red dots, and branch marking the inferred first appearance of the anchor motif is shown in red. (D) Alignment of KCNQ2 and KCNQ3 C-terminal intracellular sequences near the anchor motifs. Break (vertical black line) indicates location of 5–8 omitted, poorly conserved residues. The KCNQ2/3 anchor motif (red boxed region) is similar but non-identical to that of chordate Na_V_ genes. Otherwise no homology to the Na_V_ DII–DIII loop sequence shown in B is evident. Shading indicates conservation within the 7 KCNQ sequences aligned: shades represent ≤15, 15–30, 31–45, 46–60, 61–75, 76–90, and 91–100% conservation. (E–F) Aligned sequences at key functional sites for genes compared phylogenetically in C. Shading: grey, conserved in all KCNQ subunits; yellow, conserved in jawed vertebrate KCNQ1 subunits; red, conserved in jawed vertebrate KCNQ2-5 subunits. (E) Peptide sequence at the border of the S4-5 pore linker and the S5 pore helix, including (in KCNQ2-5 orthologues) the W residues required for retigabine interaction. (F) A portion of the tetramerization, or subunit interaction, domain. Scale bars: substitutions per residue.

**Figure 3:**
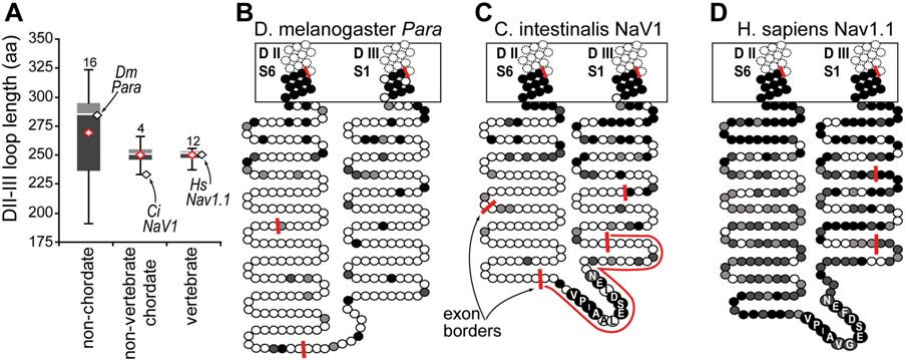
The Na_V_ channel DII–III intracellular loop is poorly conserved in invertebrates lacking the anchor motif, and highly conserved in vertebrates. (A) Plot showing lengths of DII–III loop sequences of Na_V_ channels, deduced from cDNA clones. Stick bars show range, grey boxes show 2^nd^ and 3^rd^ quartiles, and red diamond shows average length. Black diamonds show lengths of loops from species indicated. (B–D) Cartoons depicting the degree of sequence conservation and exon borders (red bars) of orthologous Na_V_ channels from *D. melanogaster (para)*, *C. intestinalis* (Nav1), and *H. sapiens* (Nav1.1) in the region between D II S6 and DIII S1. Each shaded circle is one amino acid. In non-chordates (e.g., fly), the transmembrane and very membrane-proximal portions of the intracellular loop show high conservation with vertebrates, but the remainder of the loops are poorly conserved in sequence and length. In protochordates (e.g., *C. intestinalis*), a series of highly conserved residues (VPIAAIESDLDN, residues labeled) appears on a short, novel exon (red line in C); the rest of the loop is poorly conserved like other invertebrate genes. However, the mean length of the 4 known protochordate NaV1 loops is nearly identical to those of vertebrates. Among vertebrate genes (e.g., human Nav1.1), the entire loop is more highly conserved, and has a simplified exon structure, with the anchor motif part of the same, exceptionally long exon as the conserved DII6 transmembrane segment. The shading scheme is based on alignment of the indicated sequence and six vertebrate Na_V_ channel sequences. Shading scale represents, from darkest to lightest, matching of 5–6 of 6, 3–4 of 6, 2 of 6, and 0–1 of 6 vertebrate sequences.

Na_V_ channels with rapid opening and closing kinetics are present on the motor axons and stinging nematocysts of jellyfish, where they serve in escape swimming, defense, and predation [40],[41]. Although cnidarians appear to possess only a single Na_V_ channel gene, in many protostomes and deuterostomes, multiple homologous Na_V_ channel genes derived from a common ancestor are present (e.g., *Drosophila melanogaster*, n = 2; *Ciona intestinalis*, n = 4; *Homo Sapiens*, n = 10) [1], [42]–[44]. The 10 mammalian Na_V_ genes are linked to the four mammalian *hox* loci, implying that they all descended from a single gene linked to the ancestral bilaterian *hox* locus [37],[44],[48]. Phylogenetic analysis of the origin of the anchor motif supported this scenario (Figure 2A–B and S1). All vertebrate Na_V_ channels unambiguously form a clade including a single basal chordate Na_V_ gene, called TuNa1 when first cloned and later renamed NaV1 [43],[45],[46]. NaV1 is conserved in the genomes of the tunicates *C. intestinalis*, *Ciona savignyi*, *and Halocynthia roretzi* and the cephalochordate *Branchiostoma floridae* (amphioxus). Significantly, sequence analysis revealed that these orthologous chordate NaV1 genes all inherited anchor motifs like those common to jawed vertebrates; all other invertebrate Na_V_ genes lacked any evidence of such motifs (Figure 2B, Table S1). The basal chordate anchor motifs and those in vertebrates were identically located, at a position slightly beyond the midpoint of the intracellular loop between DII and DIII (the second and third Na_V_ channel homologous domains, Figures 3 and S2). In *B. floridae* and tunicates, the Na_V_ anchors were encoded on a single short exon, and were flanked by poorly conserved sequences (Figures 3C and S2). The novel “anchor exon” was absent from protostome Na_V_ genes (e.g., Figure 3B). Whereas non-NaV1 DII–DIII loops exhibited considerable variability in both amino acid sequence and length, the chordate NaV1 and vertebrate DII–DIII loops bearing Na_V_ anchors were highly conserved in length.

The Na_V_ genes lacking anchor motifs (i.e., all non-chordate Na_V_ genes and chordate NaV2-4 genes) all appeared basal to, and exhibited greater sequence divergence than, the NaV1-like gene clade. Phylogenic relationships among these anchorless genes appeared complex, which could potentially reflect gene duplications and losses that remain unresolved (Figure 2A). For example, the fly Na_V_ gene, *Para*, appeared phylogenetically close to the chordate NaV1 genes, but lacked an anchor motif (Figure 2A). Also, *Para* is known to be unlinked to the fly *hox* locus [47], implying a genetic rearrangement in either the chordate or protostome lineage. Echinoderms are the non-chordate phylum closest to chordates (Figure 1E). The echinoderm *S. purpuratus* (sea urchin) possessed an orthologue of tunicate NaV2 genes, but no evidence for a sea urchin NaV1 orthologue was detected, suggesting gene loss. The genome of *C. elegans* lacks any Na_V_ channel gene. By contrast, vertebrate Na_V_ isoforms serving specialized fast signaling functions in brain, nerve, heart, and muscle arose from chordate NaV1 and conserved the anchor motif [44],[48],[49].

### Axon Initial Segment Na_V_ Channel Clustering Is Prominent in Lamprey

**Figure 4:**
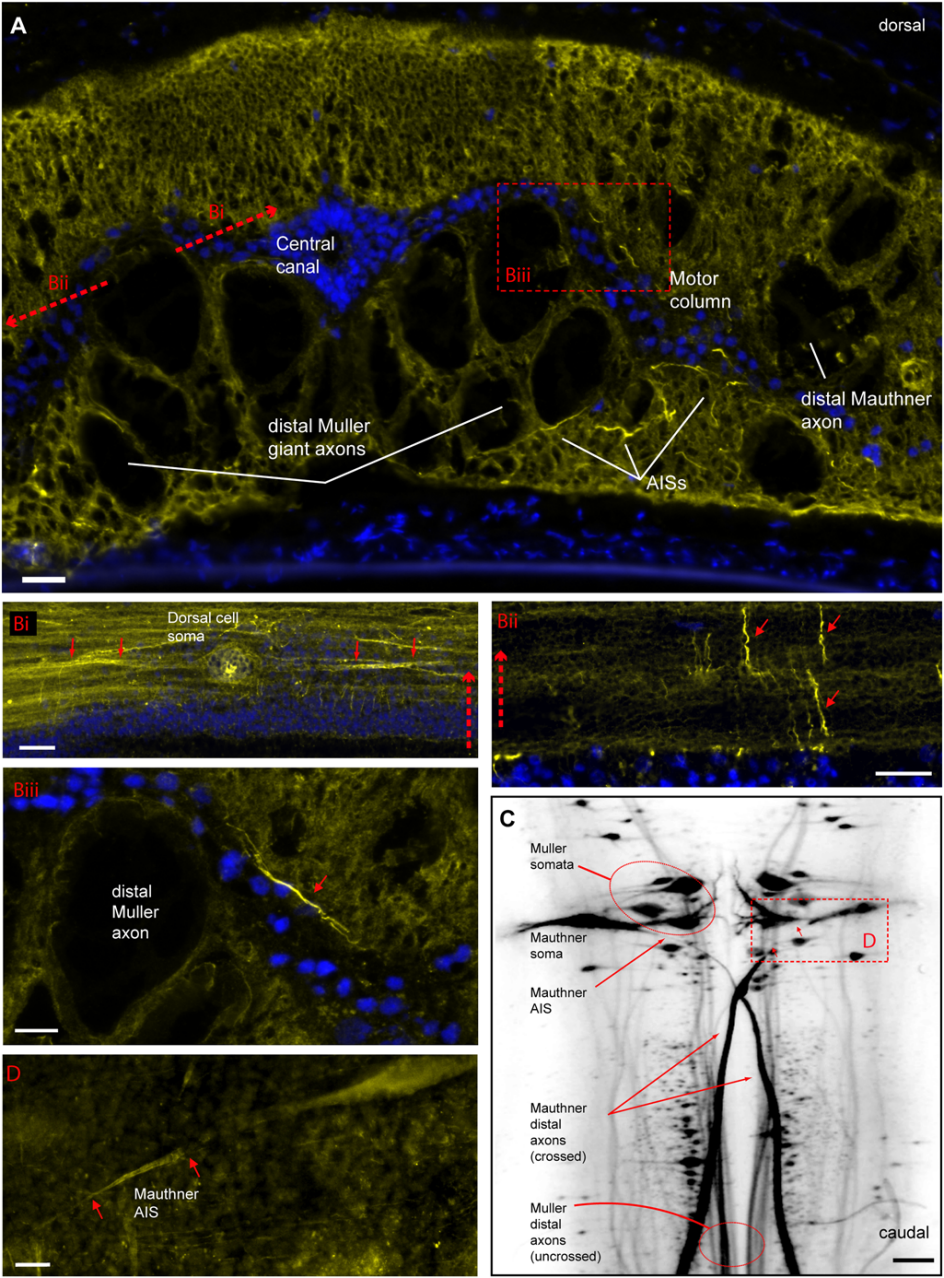
Na_V_ immunostaining of lamprey brain and spinal cord reveals linear profiles similar in appearance to mammalian AISs. (A) Transverse cryosection through lamprey spinal cord immunolabeled for Na_V_ channels (yellow). Nuclei are stained using DAPI (blue). Large distal Müller and Mauthner axons show little Na_V_ channel membrane immunolabeling, but small intensely labeled profiles have morphology suggestive of AISs, and are clustered near the motor column. Red lines and box indicate approximate location, plane and orientation of adjoining higher magnification horizontal (Bi, Bii) and transverse (Biii) section images. (Bi) Dorsal sensory neuron, with a bipolar axon. Both rostral and caudal axon branches show increased Na_V_ channel immunolabeling in their proximal portions (arrows). (Bii) AIS-like profiles are abundant in oblique horizontal sections near grey matter. (Biii) Higher magnification view of AIS-like Na_V_ channel immunostaining near motor column in spinal cord cross-section. (C) Low power view of lamprey rhombencephalon in whole mount. Reticulospinal neurons have been back-filled via their large descending axons. Somata, narrowed initial segments, and large distal axons of Müller and Mauthner cells are indicated. Box encloses the location shown at higher magnification in panel D. (D) Widefield epifluorescence image of lamprey rhombencephalon whole mount showing soma and AIS of Mauthner neuron immunolabeled for Na_V_ channels (yellow). Scale bars: A, 125 µm; Ai, 20 µm; B, Bi, Bii, 25 µm, Biii, 12.5 µm.

Lampreys are jawless vertebrates, descendants of a lineage that diverged from other crown vertebrates by the early Ordovician Period, long before the evolution of myelin and saltatory conduction [39],[50],[51]. Searching the genome of the sea lamprey *Petromyzon marinus* disclosed 2 Na_V_ channel genes, both bearing anchor motifs (Figures 2A–B). We immunostained lamprey brain and spinal cord using mouse monoclonal antibodies against the highly conserved Na_V_ channel DIII–IV loop that mediates inactivation gating [52],[53]. This revealed intense labeling of long, thin structures (∼20 by 1 µm) similar in appearance to mammalian AISs, at locations neighboring neuronal somata (Figure 4). This labeling was abolished by pre-adsorption of the antibodies with the immunogenic peptide, and staining using a second, rabbit polyclonal antibody gave identical results (Figure S3). AIS-like labeling was preserved when staining was performed on unfixed sections in the presence of 0.2–0.5% Triton-X 100. Such detergent-resistance is characteristic of mammalian AIS-resident proteins due to their association with cytoskeleton [17],[20],[54].

**Figure 5:**
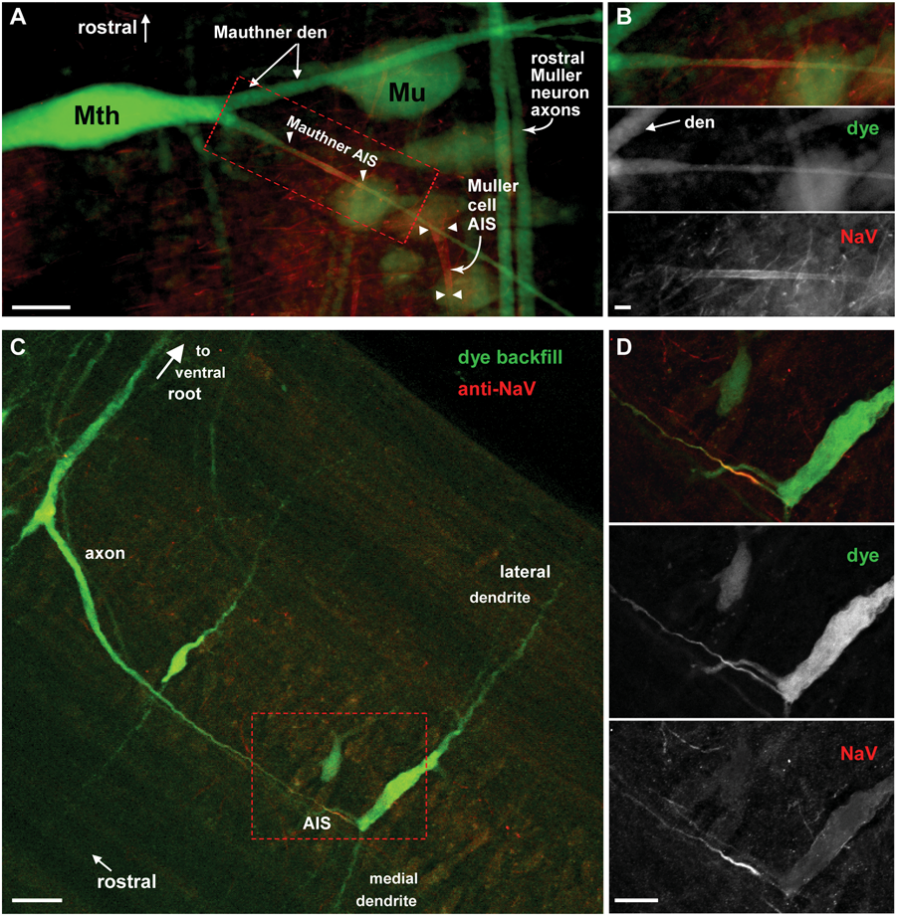
Lamprey motor system axons have narrow initial segments with Na_V_ channels clusters. (A) Detail of lamprey left rhombencephalon region whole mount showing large reticulospinal Mauthner (Mth) and Müller (Mu) neurons, backfilled via their spinal axons by *in vivo* FITC-dextran injection (green), then fixed and immunostained against Na_V_ channels (mouse Pan Na_V_, red). AISs of two Mth and Mu neurons are marked (arrowheads). (B) Higher magnification view of red-boxed region in A, showing Na_V_ channel immunolabeling at membrane of Mauthner neuron AIS. (C) Lamprey spinal cord whole mount showing several motoneurons filled in vivo via their distal axons with FITC-dextran (green), then fixed and immunostained against Na_V_ channels (red). (D) Higher magnification view of red-boxed region in C, showing dense clustering of Na_V_ channels at narrow proximal AIS of a motoneuron axon. Scale bars: 40 µm (A), 10 µm (B), 20 µm (C), 10 µm (D).

We confirmed the labeled structures to be AISs by combining immunostaining with dye-filling of identified motor system neurons [55]. In lampreys, as in jawed fish, giant Mauthner cells of the medulla project to contralateral spinal motoneurons, mediating the C-bend, a rapid escape behavior [56]. Mauthner dye-fills showed large somata and dendrites, and giant (40–80 µm diameter) distal axons, but markedly narrowed (∼5 µm diameter) proximal axons (Figure 4A, 4C, 5A). Intense membrane-associated Na_V_ channel staining was localized at the beginning of these narrowed axon initial segments (Figure 5B). The spinal motoneurons, which were previously shown by intracellular recording to initiate APs in their proximal axons [57], also exhibited patches of clustered Na_V_ channels at the beginning of narrowed AISs (Figure 5C–D). These membrane specializations, combining morphological narrowing with a high density of immobilized Na_V_ channels, would be expected to create a zone of high excitability. However, in both these neuronal types, morphological AIS narrowing was considerably lengthier than the location where channels were found at high density. Lamprey dorsal interneurons, which were shown in classical anatomical studies to lack severe narrowing at their bipolar AISs, nonetheless showed intense Na_V_ channel labeling at these sites (Figure 4Bi). Numerous other AIS-like profiles were seen in spinal cord (Figure 4Bii) and brain (data not shown). The lamprey lineage is basal to a large diversity of jawless fish taxa that, though now extinct, flourished in the Ordovician, Silurian, and Devonian Periods [50],[58]. Our molecular phylogenetic and immunostaining results suggest that in these early Paleozoic vertebrates, Na_V_ channel clustering was widely deployed as the mechanism for rapid AP initiation in the proximal axon.

### Invertebrates Possess KCNQ1 and KCNQ4/5-Like Channels That Lack Anchor Motifs

Although the five mammalian KCNQ genes are paralogues, only KCNQ2 and KCNQ3 genes possess anchor motifs (reference [20] and Figures 1B–C, 2C–D). Therefore, these motifs either evolved in an earlier KCNQ common ancestor gene, but were lost subsequently by evolution of KCNQ1, KCNQ4 and KCNQ5, or appeared first in a gene ancestral only to KCNQ2 and KCNQ3. To delineate evolutionary relationships among the KCNQ channels and the origin of the KCNQ2/KCNQ3 anchor, we reconstructed KCNQ phylogeny from known invertebrate and vertebrate sequences, as well as novel KCNQ sequences we identified from three basal chordates (*C. intestinalis*, *C. Savigyni*, *B. floridae*), a jawless fish (*P. marinus*), and a cartilaginous fish (the elephant shark, *Callorhinchus milii*). Mammalian KCNQ genes possess critical sites that confer distinctive capacities for tetramerization and drug sensitivity on non-neuronal (KCNQ1) and neuronal (KCNQ2-5) subunits (Figures 1C, 2C–F). We traced the evolutionary emergence of these sites and the anchor motif in parallel with computational phylogenetic analysis of subunit amino acid sequences.

Residues within an intracellular *subunit interaction domain* (SID) unique to the KCNQ channels (Figures 1C, 2F) dictate tetramerization rules, preventing cross-tetramerization between KCNQ1 and KCNQ2-5 subunits, and allowing some but not all combinations of KCNQ2-5 to co-assemble [59],[60]. Invertebrate KCNQ sequences fell into two groups, one with SID sequences like mammalian KCNQ1 (honey bee *Apis mellifera* KCNQ, *Caenorhabditis elegans* KQT-3, and *C. intestinalis* KCNQ1) (Figure 2F, yellow shading) and the other with sequences intermediate between KCNQ1 and the mammalian neuronal KCNQs (e.g., *D. melanogaster* KCNQ, *C. elegans* KQT-1, and the beetle *Tribolium castaneum* KCNQ). Opening of mammalian KCNQ2-5 channels by retigabine, an anticonvulsant, is associated with a conserved sequence (TAW) at a critical position within the S5 transmembrane helix that links voltage-sensor movement to the channel pore [61]–[63] (Figures 1C, 2E). In mammalian KCNQ1 channels, which are retigabine-insensitive, the TAW-equivalent position residues are TTL (Figure 2E, yellow shading). All non-chordate KCNQ genes we identified had KCNQ1-like pore-linker sequences; none had the W residue obligatory for retigabine action (Figures 2E–F). *C. elegans* possesses at least two functional KCNQ subunits, one a clear orthologue of vertebrate KCNQ1, the other grouped with chordate KCNQ2-5 genes (Figure 2C) [64]. This indicates that two ancestral KCNQ1 and non-KCNQ1 genes arose by duplication early in metazoan evolution, before the last common ancestor of arthropods, nematodes, and chordates. Non-chordate KCNQ genes most closely related to mammalian KCNQ2-5 have a similar tetramerization domain, but lack residues critical for retigabine modulation and the distal C-terminal domain that contains the anchor motif (Table S2).

### *C. intestinalis* KCNQ4/5 Has Many Properties Characteristic of Vertebrate KCNQ2-5 Subunits, but Lacks an Anchor Motif

**Figure 6:**
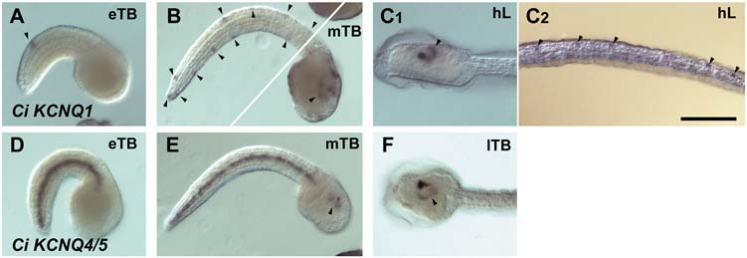
*C. intestinalis* KCNQ1 is more prominently expressed in neurons than is KCNQ4/5. Subunit mRNA expression was detected using whole mount in situ hybridization. Animals were allowed to develop at 18°C for the indicated times after fertilization in vitro, then labeled with antisense RNA probes for *C. intestinalis* KCNQ1 (A–C) or *C. intestinalis* KCNQ4/5 (D–F), and stained using NBT/BCIP. (A) At 10.5 hours post-fertilization, a pair of tail dorsal midline neurons are stained (arrowhead). (B) At 11.6 hours post-fertilization, numerous dorsal and ventral epidermal sensory neurons in tail and trunk (arrowheads, left), and labeling of the cerebral ganglion (right), is apparent. (C) At 17.2 hours post-fertilization, continued staining of central and peripheral neurons of free swimming larva is apparent C1. Strong staining of caudal portion of cerebral ganglion (arrowhead). C2. Staining of epidermal sensory neurons (arrowheads). (D) At 10.5 hours post-fertilization, KCNQ4/5 staining is strongly apparent in the notochord, but absent from central and peripheral neurons. (E) At 11.6 hours post-fertilization, strong notochord staining persists, and weaker staining of ventral cerebral ganglion is detectable. (F) At 15.5 hours post-fertilization (immediately before hatching), weak staining is detected in the posterior-ventral half of the cerebral ganglion. eTB, early tailbud; mTB, mid-tailbud; lTB, late-tailbud; hL, hatched larva. Scale bar, 100 µm.

**Figure 7:**
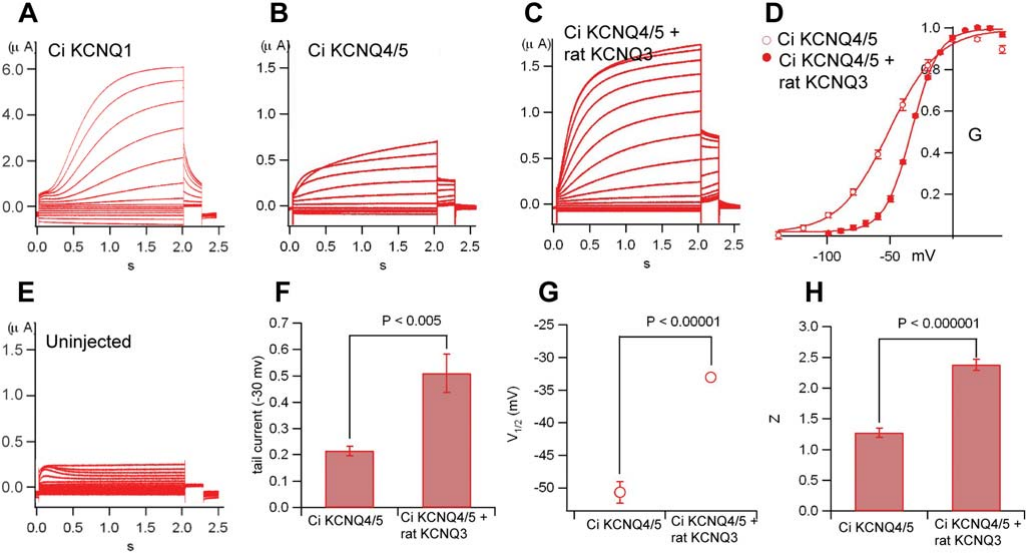
*C. intestinalis* KCNQ4/5 gives small currents in *Xenopus* oocytes, but forms heteromers with mammalian KCNQ3 that express more efficiently. (A) Family of large KCNQ1 currents elicited by voltage steps. (B) Family of small *C. intestinalis* KCNQ4/5 currents elicited by voltage steps. (C, D, F, G, H) Co-expression of *C. intestinalis* KCNQ4/5 with rat KCNQ3 results in expression of heteromeric currents with altered kinetic properties. Expression of rat KCNQ3 only resulted in currents (not shown) undistinguishable from uninjected oocytes (E). Co-expression of *C. intestinalis* KCNQ4/5 with rat KCNQ3 produced currents that were larger in amplitude than *C. intestinalis* KCNQ4/5 alone (C, F), activated at more depolarized membrane potentials (D, G), and had steeper voltage-dependence (H).

We cloned *C. intestinalis* KCNQ1 (GenBank FJ461775), and one additional gene, previously called Ci KCNQ2/3/4/5 [43], but more closely related to vertebrate KCNQ4/5 than KCNQ2/3 genes (Figures 2C, S4, S6). Ci KCNQ4/5 (GenBank FJ461778) possessed a pore-linker region of identical sequence to vertebrate KCNQ2-5 subunits, including the W required for retigabine action (Figure 2E). In situ hybridization revealed, remarkably, widespread expression of *C. intestinalis* KCNQ1 in central and peripheral neurons (Figure 6A–C). Ci KCNQ4/5 was conspicuously detected in the developing notochord, but showed minimal neuronal expression (Figure 6D–F). Ci KCNQ1 expressed robustly in *Xenopus* oocytes, generating non-inactivating currents with slow activation and deactivation (Figure 7A). Ci KCNQ4/5 also expressed currents, though at low levels only slightly above background (Figure 7B, 7E). Although mammalian KCNQ3 is unable to traffic to the cell membrane when expressed alone in these oocytes, mammalian KCNQ2, KCNQ4 and KCNQ5 can co-assemble with KCNQ3 to form heteromeric channels that traffic to the surface and conduct very robustly [65]. Ci KCNQ4/5 possesses a neuronal-type tetramerization domain (Figure 2F), and its ability to conduct was increased several-fold by coexpression with mammalian KCNQ3 (Figure 5). Such coexpression also right-shifted and steepened voltage-dependence (compared to Ci KCNQ4/5 alone, Figure 7D, F–G), indicating that Ci KCNQ4/5 can co-assemble with mammalian KCNQ3 via a functional KCNQ2/3/4/5-type tetramerization domain. Thus Ci KCNQ4/5 shares ancestry with mammalian neuronal KCNQ2-5 subunits and exhibits functional features characteristic of those subunits, even though Ci KCNQ1 is the predominant KCNQ channel in *C. intestinalis* neurons. Searches of the amphioxus genome database also revealed fragments of 2 KCNQ genes, KCNQ1 and KCNQ4/5-like (Table S1), but both these genes and the entire amphioxus genome lack sequences encoding a KCNQ-type anchor domain. In cephalochordates and tunicates, the KCNQ gene divergence leading towards the KCNQ2/3 genes had begun, but remained incomplete.

### The KCNQ2/3 Anchor Is a Shared Feature of Extant Jawed Vertebrates

**Figure 8:**
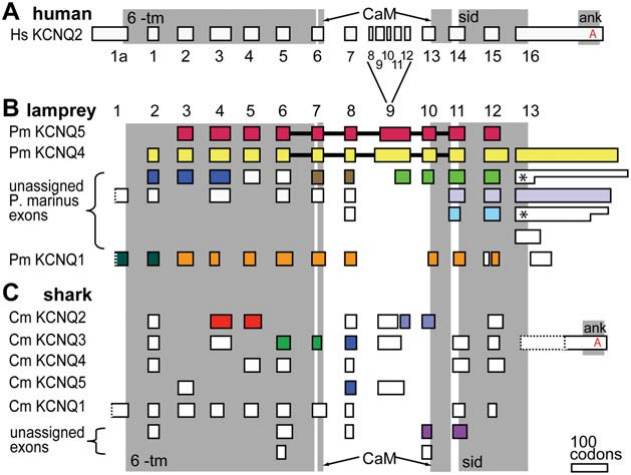
The KCNQ ankyrin-interaction domain evolved in the transition between ancestral jawless and jawed vertebrates. (A) Human (*H. sapiens*, Hs) KCNQ2 exon structure, numbered based on previous reports [107]. Grey boxes indicate locations of functionally conserved domains (6TM, the six transmembrane segments and pore region; CaM, the discontinuous calmodulin-binding IQ domain; sid, the subunit interaction domain mediating tetramerization; ank, the conserved domain containing the ankyrin-interaction motif). (B) Diagram summarizing lamprey (*P. marinus*, Pm) KCNQ genomic analysis and cDNA cloning indicating that lampreys possess KCNQ1, KCNQ5, KCNQ4, and, possibly, two additional KCNQ4-like genes. Exons (renumbered as indicated) linked *in silico* by overlapping of genomic sequencing traces are shown in identical colors. Exons linked by cDNA cloning are connected by heavy black bars. Unlinked exons are shown in white. Two different exon 1 traces had start codons that could not be determined (due to poor conservation, dotted borders). KCNQ1 exons were confirmed by reciprocal BLAST analysis versus vertebrate and invertebrate genomes. Five different non-KCNQ1 3′ exons (exon 13) were identified; two were represented in the genomic traces by sequences with different stop codon positions (asterisks). This may be the result of heterozygosity in the source genomic DNA [66]. (C) Diagram of shark (*C. milii*, Cm) KCNQ gene family as elucidated from the partially sequenced genome. Exons containing orthologues of mammalian KCNQ1 through KCNQ5, identified by reciprocal BLAST search, are indicated. One trace contained the ankyrin binding domain (distal exon 13 region) of KCNQ3.

In addition to KCNQ1, the genome of the lamprey *P. marinus* contains sequences suggesting the existence of four other KCNQ genes (Figure 8B; Table S2). Each possesses TAW sequences associated with retigabine sensitivity and non-KCNQ1-type SID regions mediating tetramerization (Figure 8, Figure 2E–F). We cloned brain cDNAs encoded by two of these genes (Figure S5). Phylogenetic analysis revealed these cloned cDNAs (GenBank FJ461777 and FJ461776) to be likely orthologues of KCNQ4 and KCNQ5 (Figure 2C, Supplementary Figure 6). Phylogenetic analysis of predicted polypeptide sequences indicated that the two remaining genes were most closely homologous to KCNQ4 (Figure 2C). Attempts to obtain cDNAs for these additional genes were unsuccessful, suggesting either developmentally or spatially restricted mRNA expression, or that they may be variant KCNQ4 alleles (heterozygosity in individual lamprey is reported to be very high, [66]). Nonetheless, sequence encoding a KCNQ-type anchor motif is absent from these predicted genes and from the entire 5.9×-redundant lamprey genome database. By contrast, although only sequenced to 1.4× redundancy (estimated 75% coverage) [67], the elephant shark genome database contains an exon encoding one KCNQ2/3 anchor motif and nearby conserved residues (Figures 2D and S7), and pairs of exons that appear orthologous to vertebrate KCNQ2 and KCNQ3 genes, respectively (Figure 8C and Table S3).

### The Na_V_ and KCNQ Anchor Motifs Appear To Be Topologically Analogous

In the chordate NaV1 and co-orthologous vertebrate Na_V_ genes, anchor motifs lie in the sodium channel intracellular loop between homologous domains II and III, at a highly conserved distance from the DIII S1 (∼97±3.1 residues) and DII S6 (113±7.9 residues) transmembrane segments (Figure S2, see Methods). The KCNQ2 and KCNQ3 anchor motifs are about 450–500 residues distant from the end of the S6 membrane helix. However, approximately the first 300 of these residues are believed to have a compact ternary structure near the membrane (Figure S7), based on mapping of conserved adjoining regions for interaction with the membrane lipid phosphatidyl inositol 4,5 bisphosphate and calmodulin, and for subunit interaction [59], [68]–[70]. Among 16 vertebrate KCNQ2 and KCNQ3 subunits, the polypeptide portion between the SID end, and the start of the conserved domain containing the anchor motif, has low sequence conservation and no known function, but a conserved length of 129±7.5 residues (Figure S7). This is similar to the conserved distance between the membrane and anchor motifs in Na_V_ channel polypeptides. Thus, Na_V_, KCNQ2, and KCNQ3 channel anchors appear to have “mooring lines” of similar, conserved length, allowing them to access ankyrin immobilized below the membrane surface (Figure 1B).

## Discussion

**Figure 9:**
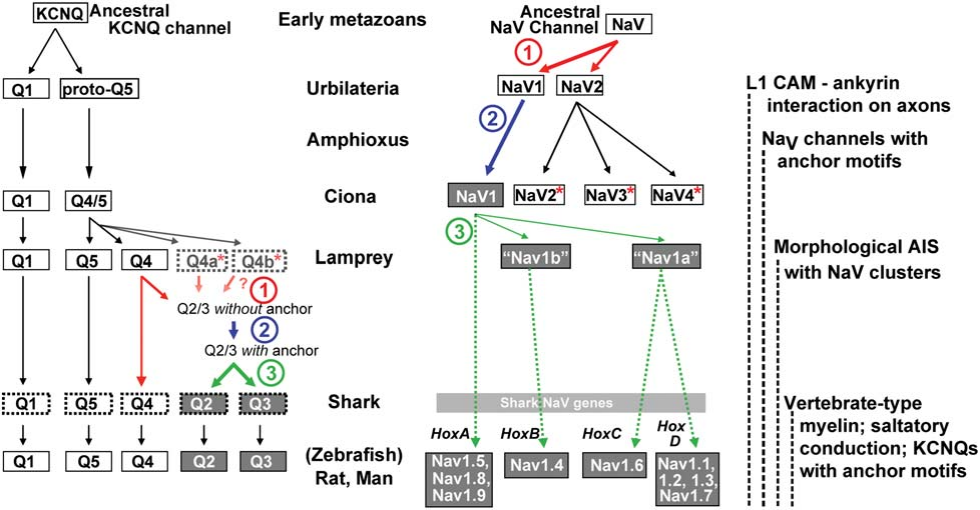
Anchor motifs evolved sequentially in Na_V_ and KCNQ channel families. Diagrams summarize the evolutionary history of KCNQ channels (left), Na_V_ channels (right), and their anchor motifs. In each gene family, three steps are highlighted: (step 1, red arrows) gene duplication preceding appearance of the anchor, (step 2, blue arrows) evolution producing the anchor motif, and (step 3, green arrows) additional duplication resulting in parologues conserving the motif. Representative species studied are listed in the center. Genes possessing anchor motifs are shaded grey. The Na_V_ channel motif arose before the common ancestor of amphioxus and tunicates. In KCNQ channels, an inferred KCNQ2/3 gene acquired the motif, after lamprey but before the duplication producing shark KCNQ2 and KCNQ3. Where 3 or more genes are shown arising from an ancestor gene, an unresolved sequence of gene duplications (i.e., polytomy) is present. Genes apparently lacking orthologues in more recently evolved phyla are indicated by asterisks. Genes identified genomically without cDNA confirmation have dashed border boxes. Lamprey KCNQ4a/b genes are drawn lightly, indicating their uncertain status (see Results). Shark Na_V_ genes (not characterized in this study) are omitted. Hox-linked vertebrate Na_V_ genes underwent lineage-specific genome duplications, as indicated by boxed gene groups. Associated hox clusters are labeled [44],[48]. Ankyrin interaction with L1 CAMs on axons evolved before the deuterostome-protostome divergence [75]–[78].

In many mammalian neurons, clustering of ion channels at the AIS and nodes of Ranvier is the basis for rapid, reliable, and precisely-timed action potential initiation and conduction [3], [11]–[14]. Our investigation of the evolutionary origin of this clustering yielded three main findings (Figure 9). First, evidence of inheritance of the Na_V_ channel anchor motif is present in the earliest-diverging extant chordate (amphioxus), as well as in multiple ascidians, indicating this motif appeared at least before the last common ancestor of living chordates, in the early Cambrian Period. Second, clustering of Na_V_ channels at narrow AISs is present in lamprey, an early agnathan, indicating that this specialization mediating AP initiation was present long before myelin and nodes of Ranvier evolved. Third, signals for clustering KCNQ channels appeared considerably later than in Na_V_ channels, after sequential gene duplications that first yielded KCNQ4 and KCNQ5, then the inferred common ancestor gene, KCNQ2/3. The KCNQ2/3 gene appears absent in lamprey. In shark–the next available model organism after lamprey and earliest of extant jawed vertebrates–KCNQ2 and KCNQ3 paralogues are both present. Thus, the Na_V_ and KCNQ anchors both evolved in recently duplicated genes (Figure 2; Figure 9, red arrows), exemplifying the important principal that relaxed selection experienced by paralogues after their birth affords transient opportunity for evolutionary innovation [72],[73]. The specific evolutionary mechanisms in evidence include both subfunctionalization (i.e., the restriction of expression of duplicated channel genes to neural and non-neural cells) and neofunctionalization (i.e., the evolution of new intracellular domains bearing the anchor motifs) [74].

### Multiple Functions of Na_V_-Ankyrin Interaction: Inward Current Density Elevation, Capacitance Reduction, Cell Polarization

Ankyrins have earlier-evolved roles on axons, predating the divergence of arthropods, nematodes, and chordates, which, though incompletely understood, include the mediation of L1-family cell adhesion molecule (L1-CAM) signals for pathfinding, cell-cell interaction, and synaptogenesis [75]–[78]. L1-CAMs of fly, worm, and vertebrates share a conserved intracellular ankyrin-binding motif, FIGQY, required for these functions. *C. intestinalis* possesses one ankyrin gene, ancestral to the three vertebrate ankyrin paralogues [45],[79]. The evolutionary co-optation of axonal L1-CAM/ankyrin/spectrin/actin complexes for clustering of Na_V_ channels resulted in several new advantages. Because rapid AP propagation depends on a low ratio of membrane capacitance to axial conductance, invertebrates lacking myelin rely on large diameter axons to increase conductance speed [3]. However, initiation of APs in such giant axons is necessarily slowed, since the rate of depolarization from rest is dependent on membrane capacitance, and therefore, axonal circumference at the initiation site. The spectrins are large, extensible molecules that can be linked into a submembranous network by short filamentous actin hubs [15],[80]. Where Na_V_ channels are linked by dense spectrin-actin networks, local inward conductance density can be very markedly elevated [13]. Furthermore, spectrin behaves as a molecular spring that preferentially adopts conformations about half its fully extended length, a property which contributes to erythrocyte mechanical resiliency [80]. At nodes of Ranvier, spectrin shortening appears to function like a corset, constricting the diameter overlying axonal membrane [16], simultaneously reducing the total membrane capacitance and increasing channel density. Placing this molecular complex in the AIS provides very rapid depolarization at this location, and thus, precise spatiotemporal control of initiation [14]. Finally, in erythrocytes, epithelial cells, and mammalian axons, actin-spectrin networks and ankyrin-bound transmembrane proteins form a dense barrier that retains proteins bound within and excludes non-bound proteins, thereby helping maintain subcellular domains containing distinctive populations of proteins and lipids [15],[81]. Thus, achieving strongly preferential AP initiation at the AIS through this mechanism divides the neuron into distinct upstream (somatodendritic) and downstream (axonal) domains, both morphologically and functionally [13],[14].

### Voltage-Gated Sodium Channel Clusters as “Excitozones”

In clarifying the evolutionary relationship between channel clustering at the AIS and at the unmyelinated gap in the node of Ranvier, our studies highlight the need for clearer distinction between the membrane-associated protein complexes themselves and these two axonal subcellular domains. Although Na_V_ channel-interacting complexes are conspicuous at vertebrate AISs and nodes, these subcellular locations contain multiple additional components (e.g., AISs have synapses, fasciculated microtubules, and cisternal organelles; nodes have paranodal septate-like junctions, etc.). Also, Na_V_ channel complexes have recently been found in mammals at sites of AP initiation and reinitiation other than AISs and nodes, including at the afferent endings of sensory nerve fibers, the dendrites of olfactory bulb neurons, and cell-cell junctions in cardiomyocytes [33],[82],[83]. Finally, the axons of protostomes must possess a point of origin, and thus have “axonal initial segments.” Because discussion is hampered by lack of adequate terminology, we suggest that this crucially important, chordate-specific membrane-associated complex, i.e., Na_V_ channels clustered via ankyrin and cytoskeletal interaction, be called the excitozone, which is succinct. The excitozone is a not a particular subcellular domain, but a modular (and therefore, scalable and pluripotent) membrane-cytoskeletal assemblage, deployed at a variety of locations on vertebrate (and, possibly, invertebrate chordate) excitable cells for AP generation and regeneration.

### Why Do Na_V_ and KCNQ2/3 Channels Bear Similar Anchor Motifs?

Although the Na_V_ and KCNQ2/3 anchor sequences are very similar, they are non-identical. Within the motifs, 7 of 10 residues implicated in ankyrin interaction are shared [17],[18],[20]. These motifs are contained within longer sequences that are highly conserved within the respective vertebrate Na_V_ and KCNQ2/KCNQ3 genes, but completely distinctive between the two channel families (Figures 2B, 2D, S2, S7). Three mechanisms might allow KCNQ genes to acquire anchors subsequent to the appearance of similar motifs in Na_V_ channels: transfer of the Na_V_ sequence by retrotransposition and subsequent divergence, transfer without retrotransposition (e.g., exon shuffling) and divergence, or convergent evolution [84]. The first two mechanisms would make the Na_V_ and KCNQ anchors homologous, i.e., derived from common ancestral DNA. Under the third, the motifs would be independently evolved, i.e., analogous or homoplasic. KCNQ2 and KCNQ3 gene sequences encoding the anchors lie near the 3′ end of exceptionally long exons (Figure 8, Supplementary figure S7). Because the 5′ portions encode subunit interaction domain sequences absolutely required for channel function [59], these exons cannot be lost, but their 3′ vary widely in sequence and length in KCNQ4 and KCNQ5 genes. By mutation, the inferred common ancestor gene, KCNQ2/3, might have acquired a sequence weakly analogous to the Na_V_ anchor at the 3′ end of this obligatory exon, causing these channels to first be retained at excitozones. Natural selection based on the physiological advantages conferred by colocalization of Na_V_ and KCNQ channels, and partial sequence convergence, is a plausible alternative mechanism to transfer of the preexisting Na_V_ motif and divergence. Although examples of functional convergence are common in biology, we are unaware of convergence between unrelated proteins occurring simultaneously at the level of amino acid sequence, molecular mechanism, localization, and function [36], as may have occurred in this instance.

Each ankyrin-G molecule possesses one docking site for interaction with the Na_V_ anchor motif [33]. The high sequence similarity in KCNQ and Na_V_ anchors suggests they compete for these ankyrin-G sites, thereby conferring precise control of the number and ratio of the two channel types at AISs and nodes. Voltage-clamp studies show a 40∶1 ratio of Na_V_ and KCNQ conductance at mammalian peripheral nodes of Ranvier [85]. However, because KCNQ channels have a higher open probability than transient Na_V_ channels in the voltage range between resting membrane potentials and AP threshold, and close very slowly once opened by depolarization, a small proportion of KCNQ channels can significantly dampen excitability [25],[27],[86]. The mechanism setting the excitozone Na_V_∶KCNQ channel ratio, and its potential for plasticity, deserves further study. The critical importance of this ratio is illustrated by mutations that disrupt the function of the AIS-localized Na_V_ and K_V_ channels in humans and transgenic mice, causing conspicuous neurological phenotypes: myokymia, neuromyotonia, episodic ataxia, and epilepsy [24], [29], [87]–[90].

### The Excitozone and the Divergence and Success of Vertebrates

The excitozone has evolved, in its components, cellular distribution and function, in parallel with the chordates. The localization of Na_V_ channels in *B. floridae* and *C. intestinalis* neurons is unknown. Recent morphological studies have shown that many of the neurons of *C. intestinalis* have polar morphology of the vertebrate type, with long, branched dendrites or afferent endings that converge upon somata, and a single axon arising from the soma and innervating the efferent targets [91] (i.e., Figure 1A, though without myelin). However, no *C. intestinalis* neurons exhibit conspicuously narrowed AISs [91]. Although rapid conduction may not be required given the small size (∼1 mm) and relatively modest behavioral repertoire of short-lived (1 day) planktonic *C. intestinalis* larvae, it will be interesting to learn if excitozones contribute to AP initiation, either in sensory afferents or efferent AISs. Compared to *C. intestinalis* larvae, ancient jawless fish were larger and longer-lived, and engaged in far more rapid and complex behavior [92]. The presence of both Na_V_ channel clustering and axonal diameter narrowing at AIS in lampreys, extant representatives of a very basal jawless vertebrate group, indicates that these AP initiation mechanisms were well-established during the Ordovician through Devonian agnathan heyday. Sharks and other jawed fish possess myelin, and co-clustered spectrin and Na_V_ channels have been demonstrated at teleost nodes of Ranvier [93]. Our phylogenetic evidence strongly suggests the additional presence of KCNQ channels. Although only the inferred ancestor gene KCNQ2/3 evolved an anchor motif so similar in sequence to that of Na_V_ channels, further studies will likely show how other channels and regulatory proteins resident in or interacting with mammalian excitozones (e.g., MAGUKs, Kv1 channels, Iκ-Bα, see [11], [94]–[96]) evolved their own localization mechanisms. Analysis of fossil cranial nerve foramina suggests that rapid saltatory conduction probably appeared in the interval between armored but jawless, primarily bottom-feeding osteostraci and predatory placoderms with hinged and toothy jaws [51],[58]. Although many issues remain for future work, it is already apparent that the intricately interwoven structure and mechanisms of the vertebrate myelinated axon illustrates not irreducible complexity, but instead, the outcome of a series of incremental evolutionary steps.

Thus, our findings indicate that the clustering of Na_V_ channels on early chordate axons was a pivotal innovation, preceding and making possible the subsequent evolution of mechanisms for compact, energetically-efficient, rapid, and reliable AP initiation and conduction deployed by all extant jawed vertebrates [3],[14]. This conclusion represents an addendum to the influential “new head” hypothesis linking neural crest and ectodermal placode evolution to vertebrate origins and success [92],[97],[98], complementing ongoing studies of systems level morphological reorganization and its genetic control [99],[100] with a new focus on subcellular, intrinsic, neuronal electrical signaling. The new head required more elaborate mechanisms for sensation (e.g. eyes and ears), neural computation, and behavior (e.g., improved motor control and jaws). Evolution and deployment of the excitozone proceeded in parallel with and enabled a cascade of related changes integral to the new head. Localizing preferential AP initiation to a single neuronal site at the AIS conferred new polarity, uniformity, and robustness to signaling by individual neurons [13],[14]. This reorganization of the neuron ultimately allowed for signaling both by active dendritic AP back-propagation and axonal saltatory conduction. Integration of such neurons in larger networks of interconnected circuits made possible the more diverse, active, and sometimes predatory behavior of vertebrates, and a new ecological order [101]. This view of the excitozone, as an evolutionary “watershed” [102],[103], can be tested by further analysis of the distribution and function of excitozones in basal chordates and vertebrates.

## Methods

### Identification of Na_V_ and K_V_ Channel Sequences

Complementary DNAs for *C. intestinalis* KCNQ1 and KCNQ5 clones were amplified by a combination of PCR, 3′ RACE, and 5′ RACE, using a full-length cDNA pool derived from hatched larvae. To identify KCNQ channel sequences, the *P. marinus* NCBI WGS trace archive and Ensembl Pre assembly were searched using mammalian and *C. intestinalis* KCNQ channel sequences. To identify Na_V_ and KCNQ sequences from *S. purpuratus* (sea urchin), *B. floridae* (amphioxus), and *Callorhinchus milii* (elephant shark), databases at NCBI and the Elephant Shark Genome Project website (http://esharkgenome.imcb.a-star.edu.sg/) were similarly searched. Genomic DNA hits were translated and aligned using CLUSTAL to identify exon-intron junctions.

### In Situ Hybridization and *Xenopus* Oocyte Expression

Adult *C. intestinalis* were collected at Nishiura port in Gamagori (Aichi, Japan). *C. intestinalis* ova were fertilized in vitro and subjected to whole mount in situ hybridization, mounted and imaged under differential interference contrast optic using a Zeiss Axioplan microscope. *Xenopus* oocytes were isolated, cRNA prepared and injected, and two to five days later, two electrode voltage-clamping was performed as described previously [104].

### Immunostaining

Lampreys were obtained from streams feeding Lake Michigan, and housed and handled according to procedures approved by the University of Pennsylvania Animal Use and Care Committee. Lamprey brain and spinal cord cryosections were prepared without fixation as described previously [20], and stained for Na_V_ channels using either mouse monoclonal (Sigma) or affinity-purified rabbit polyclonal (Millipore) antibodies against the conserved Na_V_ channel DIII–IV intracellular loop. Peptide pre-absorption control experiments were performed as described [105]. Prior to whole mount immunostaining, reticulospinal neurons were backfilled by surgically exposing and transecting the spinal cord at the level of the 4th gill slit, and inserting a gelfoam piece soaked in 5% FITC-dextran solution in PBS (10,000 Da; Invitrogen). Spinal motoneurons were backfilled by injecting dorsal muscle with FITC-dextran using a 25 gauge needle. Two to five days later, the central nervous system was removed, fixed for 30 min using 4% paraformaldehyde, and then immunostained using the monoclonal antibody, Pan Na_V_. Stained samples were imaged by widefield immunofluorescence microscopy (Nikon E80i, KE Spot 740 cooled CCD camera and Spot 4.0 software) or confocal microscopy (Leica SP2).

### Sequence Comparisons and Phylogeny Construction

Sequences were aligned using the Clustal algorithm using MEGA V4.0 [106], and adjusted manually. Phylograms and bootstrap values were calculated using minimal evolution, maximal parsimony, and neighbor joining algorithms. Calculations of mean (±S.D.) Na_V_ DII–DIII linker and KCNQ C-terminal sequence lengths, and distances between transmembrane segments, tetramerization domains, and anchor motifs, were based on genes (n = 16, each group) for which cDNA sequences were available.

## Supporting Information

Figure S1 Alternative algorithms give similar NaV channel phylogenies. Figure 2A shows NaV channel phylogeny resulting from minimal evolution algorithm. As shown here, analysis using maximum parsimony (A) or neighbor joining (B) algorithms results in very similar phylogenies.
(0.20 MB TIF)

Figure S2 Location of anchor motifs in the NaV channel DII–III intracellular loop. (A) Cartoon showing NaV channel topology. The four homologous domains (I–IV), each with 6 transmembrane segments, and the DII–III loop (shaded) are labeled. (B) Sequence alignment of 12 non-chordate, chordate and vertebrate DII–III loops. The locations of the conserved distal DII S6, anchor motif, and proximal DIII S1 segments are indicated.
(1.81 MB TIF)

Figure S3 Lamprey AISs are immunolabeled by two different NaV channel antibodies. (A) Alignment of the sp-19/Pan NaV epitope used for antibody generation [52],[53] with lamprey sequences. (B) Unfixed transverse cryosection of lamprey spinal cord, immunostained with affinity purified mouse monoclonal Pan NaV primary antibodies and Cy3-conjugated secondary antibodies (false colored yellow). DAPI (blue) shows location of cell nuclei in grey matter region of the cord. (C, D) Monochrome display of sections processed in parallel, stained using primary antibodies that were preincubated for 1 hr. with (D) and without (C, same section shown in color in B) a 25-fold molar excess of the synthetic peptide immunogen. In D and C (unlike B), image intensities have been increased linearly and identically to reveal the weakest detectable staining. As a result, B best shows selective labeling of putative AISs in locations adjoining neuronal cell bodies, C reveals saturated AIS profiles and examples of higher-than-background labeling continuing (in putative axons) beyond AISs, and D shows that both AIS and weaker axonal labeling is undetectable after peptide preadsorption. For B–D, mouse primary antibodies were detected with affinity purified, species preadsorbed Cy3-conjugated anti-mouse IgG secondary antibodies. (E) Unfixed transverse cryosection of lamprey spinal cord, immunostained with affinity purified rabbit polyclonal (sp-19) primary antibodies and affinity purified, species preadsorbed Cy3-conjugated donkey anti-rabbit IgG secondary antibodies (false colored yellow). DAPI (blue) shows location of cell nuclei. AIS profiles identical to those seen using monoclonal Pan NaV are detected.
(1.86 MB TIF)

Figure S4 Sequence alignment of *C. intestinalis* KCNQ1 and KCNQ5 with orthologous human genes. Full length *C. intestinalis* KCNQ1 and KCNQ5 sequences were obtained by PCR using primers derived from the partial genomic sequence, followed by 3′ RACE and 5′ RACE to identify start and stop codons and the polyA tract. Deduced sequences are shown aligned with human KCNQ1 and KCNQ5. Locations of functional domains of the polypeptides are indicated.
(0.90 MB TIF)

Figure S5 Alignment of derived sea lamprey and human KCNQ4 and KCNQ5 polypeptide sequences. (A) Cartoon depiction exon structure of *P. marinus* KCNQ4 and KCNQ5, deduced by cDNA cloning (colored boxes connected by black bars, limits marked by blue arrows) and genomic contigs (unlinked exons). (B) Alignment of human and *P. marinus* genes. Functional regions are labeled, and limits of cDNA clones are marked by arrows as in A.
(1.34 MB TIF)

Figure S6 KCNQ gene family phylogeny (minimal evolution) based on analysis of exons 4–14. Nodes are labeled by bootstrap values, scale indicates changes per residue. The branch on the phylogram in which the anchor motif first evolved is shown in red. Nodes associated with gene duplications are indicated by red dots. Results are similar to those derived from analysis of conserved exons 5–7 only (shown in Figure 2C). *C. intestinalis* and *P. marinus* KCNQ genes cloned here (red text) appear orthologous to KCNQ1, KCNQ5, and (*P. marinus*) KCNQ4.
(0.26 MB TIF)

Figure S7 KCNQ exons encoding the C-terminal region begin with conserved sequence encoding the subunit interaction domain, but are otherwise poorly conserved in length and sequence except for the domains of KCNQ2 and KCNQ3 bearing the anchor motif. Aligned peptide sequences corresponding to the entire 3′ coding exons of 15 representative vertebrate and invertebrate KCNQ genes are shown. Except for the initial ∼15 residues (forming part of the subunit interaction domain), only the distal domains containing anchor motifs, which are exclusive to jawed vertebrate KCNQ2 and KCNQ3 sequences (blue lettered subunits), are conserved. Codon lengths for the exons are given at bottom right; the 5′ portion of sequence for shark is unknown.
(2.67 MB TIF)

Table S1 Database of NaV and KCNQ channel genes used in this study.
(0.14 MB XLS)

Table S2 Lamprey exon sequences identified in this study. Sequences were identified by BLAST search of the NCBI whole genome database and Ensembl contig database. Sequences of exons linked by cDNA cloning, or in silico by genomic DNA assembly, are enclosed in same-colored boxes.
(0.02 MB XLS)

Table S3 Shark KCNQ channel exon sequences identified in this study. Sequences were identified by BLAST search of the Elephant Shark Genome Project (http://esharkgenome.imcb.a-star.edu.sg/) database. Tentative orthologies were assigned by BLAST of mammalian database with identified shark exons. Sequences of exons linked by genomic assembly are enclosed in same-colored boxes.
(0.02 MB XLS)
