# Supplementary figures and images for: Ion Channel Clustering at the Axon Initial Segment and Node of Ranvier Evolved Sequentially in Early Chordates
Source: PLoS Genet. 2008 Dec 26;4(12):e1000317. doi: 10.1371/journal.pgen.1000317 (PMC2597720; doi:10.1371/journal.pgen.1000317)

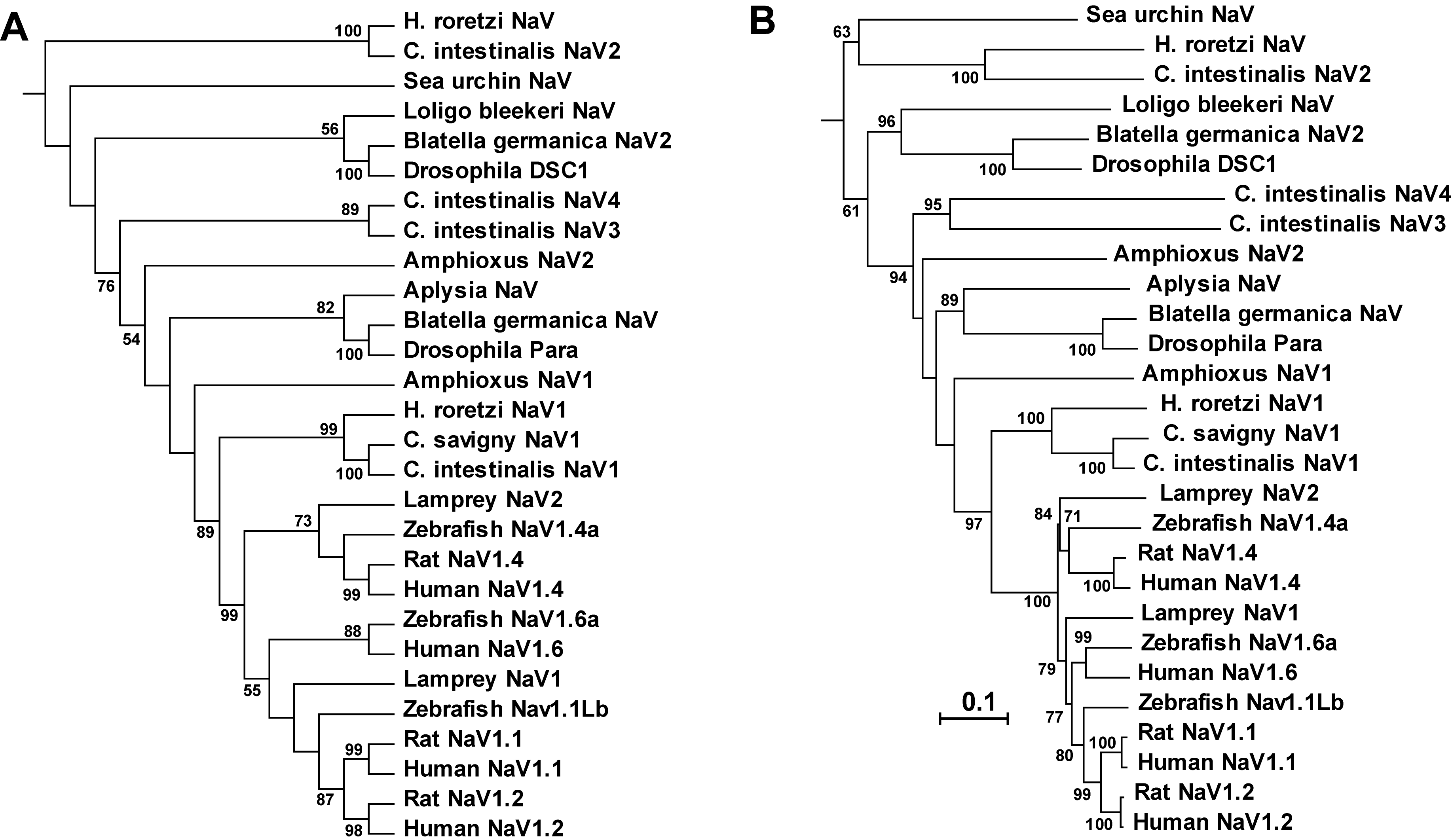

Supplement: Figure S1 — Alternative algorithms give similar NaV channel phylogenies. Figure 2A shows NaV channel phylogeny resulting from minimal evolution algorithm. As shown here, analysis using maximum parsimony (A) or neighbor joining (B) algorithms results in very similar phylogenies. (0.20 MB TIF) [file pgen.1000317.s001.tif]

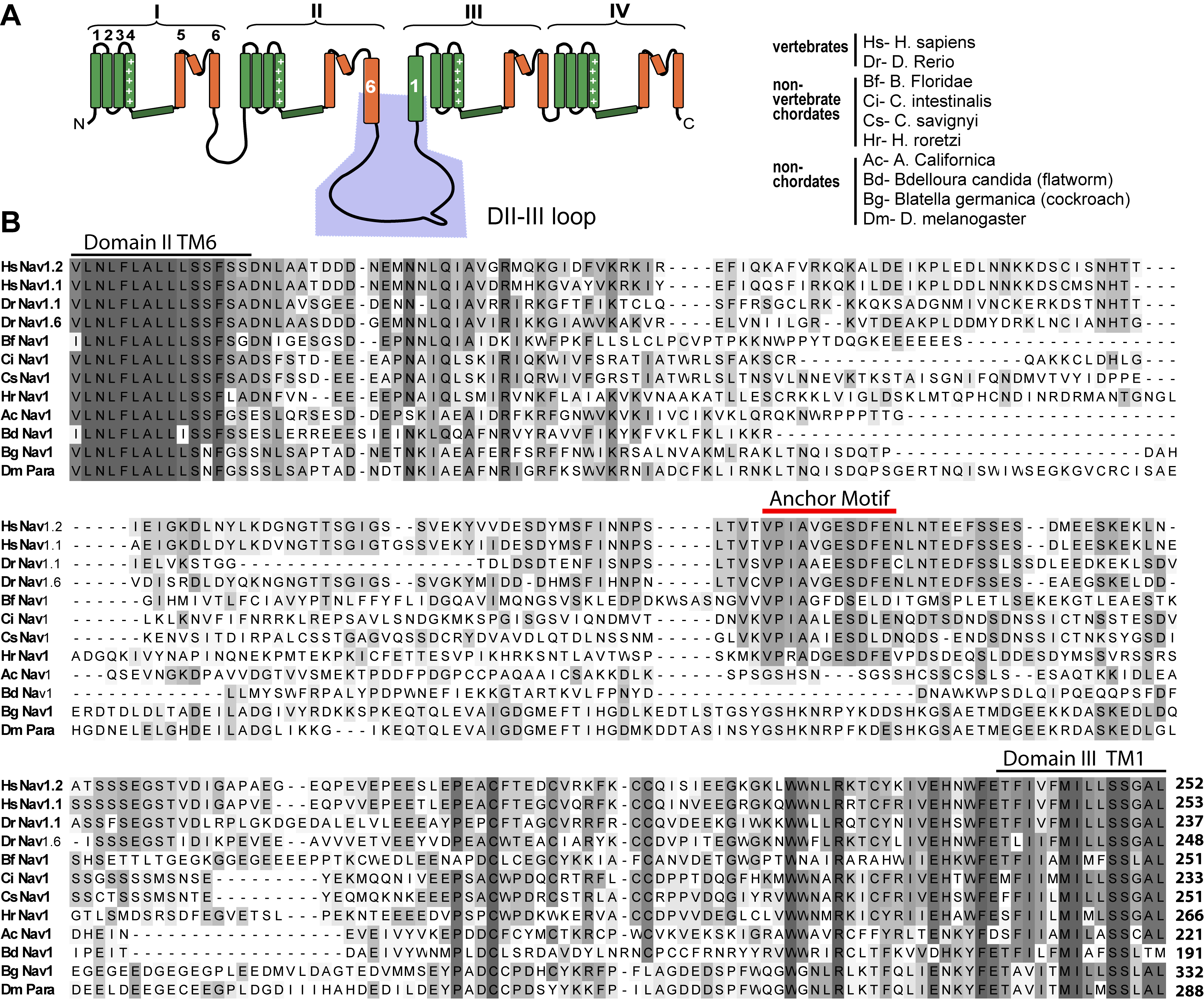

Supplement: Figure S2 — Location of anchor motifs in the NaV channel DII–III intracellular loop. (A) Cartoon showing NaV channel topology. The four homologous domains (I–IV), each with 6 transmembrane segments, and the DII–III loop (shaded) are labeled. (B) Sequence alignment of 12 non-chordate, chordate and vertebrate DII–III loops. The locations of the conserved distal DII S6, anchor motif, and proximal DIII S1 segments are indicated. (1.81 MB TIF) [file pgen.1000317.s002.tif]

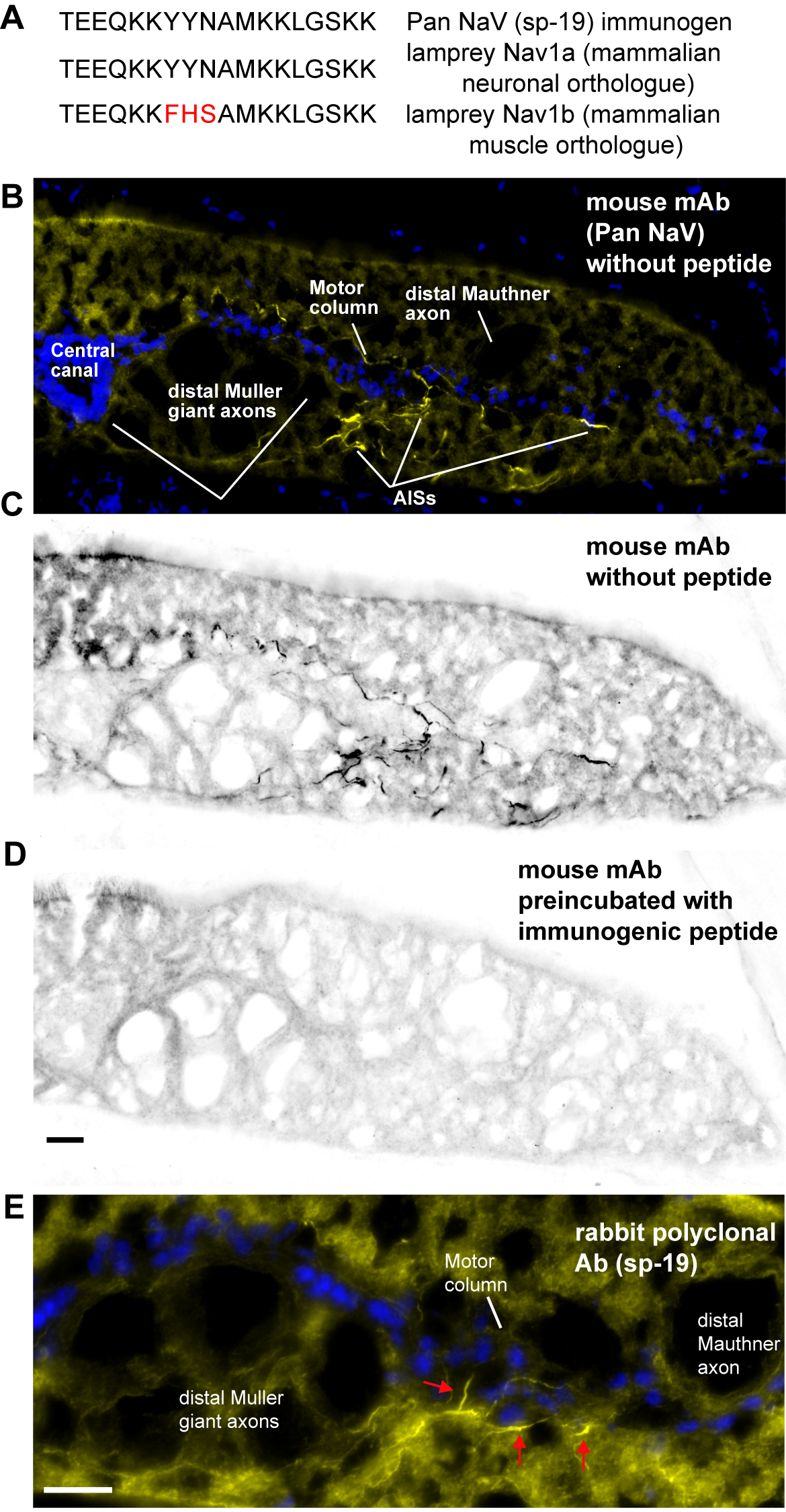

Supplement: Figure S3 — Lamprey AISs are immunolabeled by two different NaV channel antibodies. (A) Alignment of the sp-19/Pan NaV epitope used for antibody generation [52],[53] with lamprey sequences. (B) Unfixed transverse cryosection of lamprey spinal cord, immunostained with affinity purified mouse monoclonal Pan NaV primary antibodies and Cy3-conjugated secondary antibodies (false colored yellow). DAPI (blue) shows location of cell nuclei in grey matter region of the cord. (C, D) Monochrome display of sections processed in parallel, stained using primary antibodies that were preincubated for 1 hr. with (D) and without (C, same section shown in color in B) a 25-fold molar excess of the synthetic peptide immunogen. In D and C (unlike B), image intensities have been increased linearly and identically to reveal the weakest detectable staining. As a result, B best shows selective labeling of putative AISs in locations adjoining neuronal cell bodies, C reveals saturated AIS profiles and examples of higher-than-background labeling continuing (in putative axons) beyond AISs, and D shows that both AIS and weaker axonal labeling is undetectable after peptide preadsorption. For B–D, mouse primary antibodies were detected with affinity purified, species preadsorbed Cy3-conjugated anti-mouse IgG secondary antibodies. (E) Unfixed transverse cryosection of lamprey spinal cord, immunostained with affinity purified rabbit polyclonal (sp-19) primary antibodies and affinity purified, species preadsorbed Cy3-conjugated donkey anti-rabbit IgG secondary antibodies (false colored yellow). DAPI (blue) shows location of cell nuclei. AIS profiles identical to those seen using monoclonal Pan NaV are detected. (1.86 MB TIF) [file pgen.1000317.s003.tif]

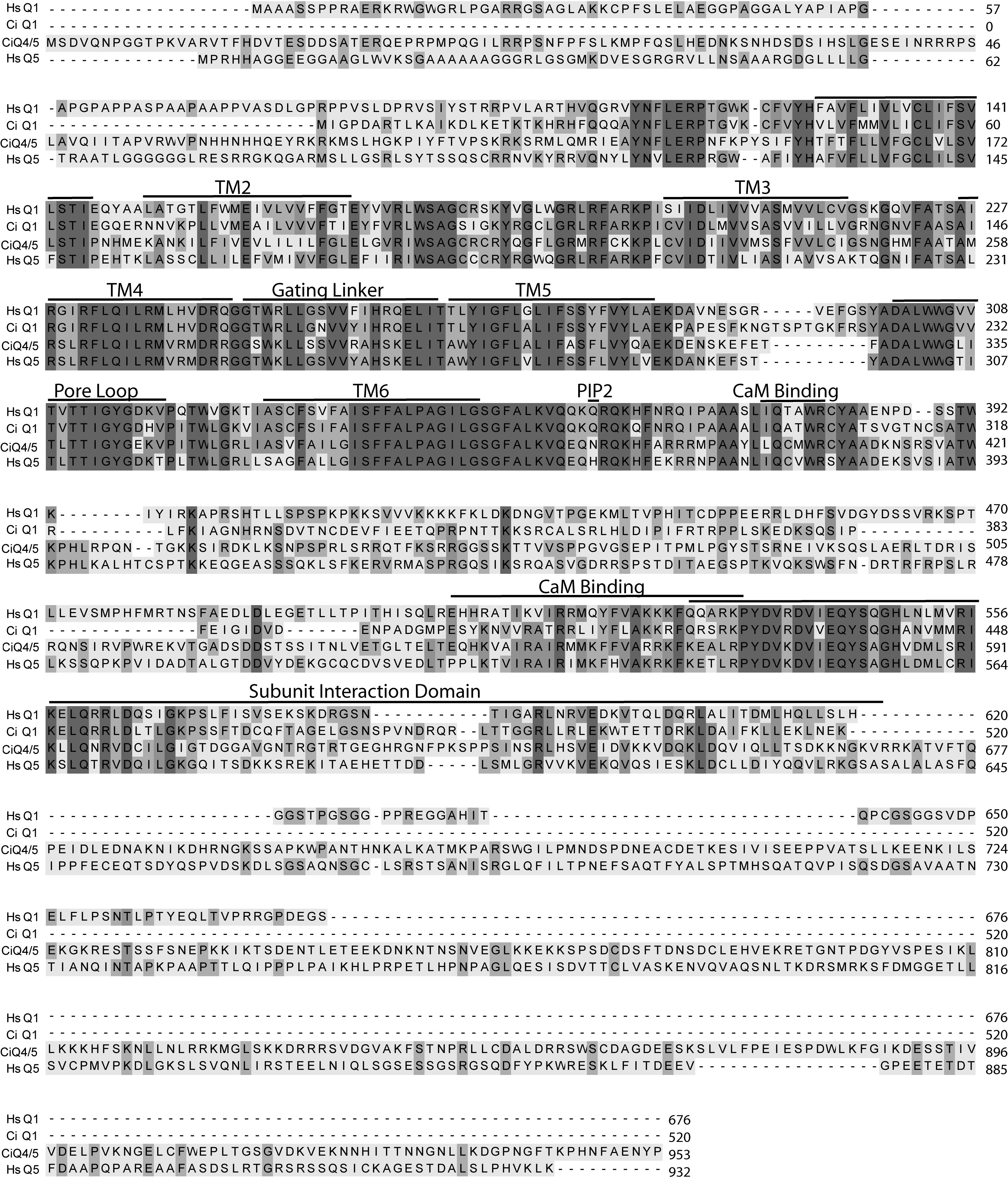

Supplement: Figure S4 — Sequence alignment of C. intestinalis KCNQ1 and KCNQ5 with orthologous human genes. Full length C. intestinalis KCNQ1 and KCNQ5 sequences were obtained by PCR using primers derived from the partial genomic sequence, followed by 3′ RACE and 5′ RACE to identify start and stop codons and the polyA tract. Deduced sequences are shown aligned with human KCNQ1 and KCNQ5. Locations of functional domains of the polypeptides are indicated. (0.90 MB TIF) [file pgen.1000317.s004.tif]

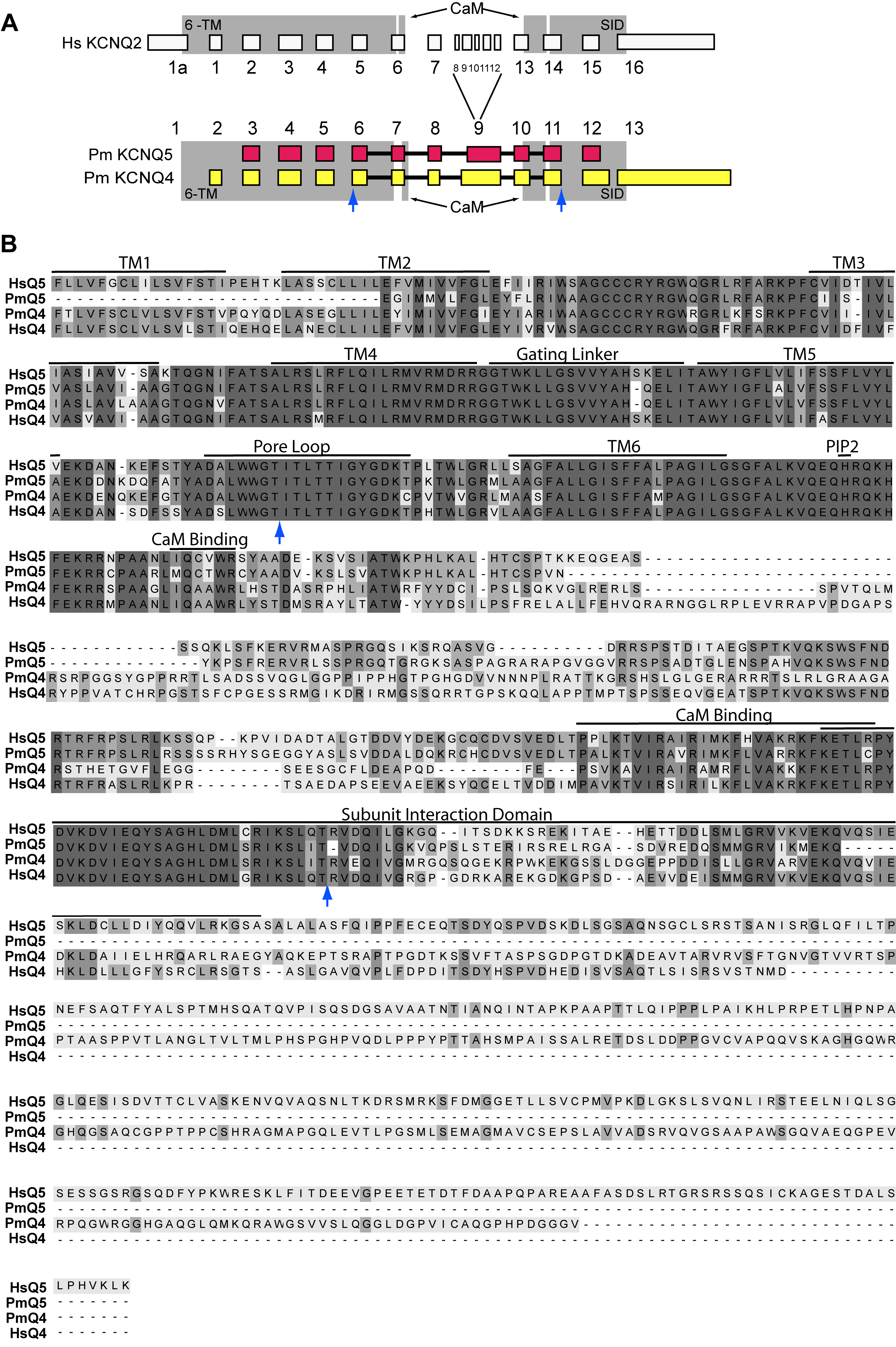

Supplement: Figure S5 — Alignment of derived sea lamprey and human KCNQ4 and KCNQ5 polypeptide sequences. (A) Cartoon depiction exon structure of P. marinus KCNQ4 and KCNQ5, deduced by cDNA cloning (colored boxes connected by black bars, limits marked by blue arrows) and genomic contigs (unlinked exons). (B) Alignment of human and P. marinus genes. Functional regions are labeled, and limits of cDNA clones are marked by arrows as in A. (1.34 MB TIF) [file pgen.1000317.s005.tif]

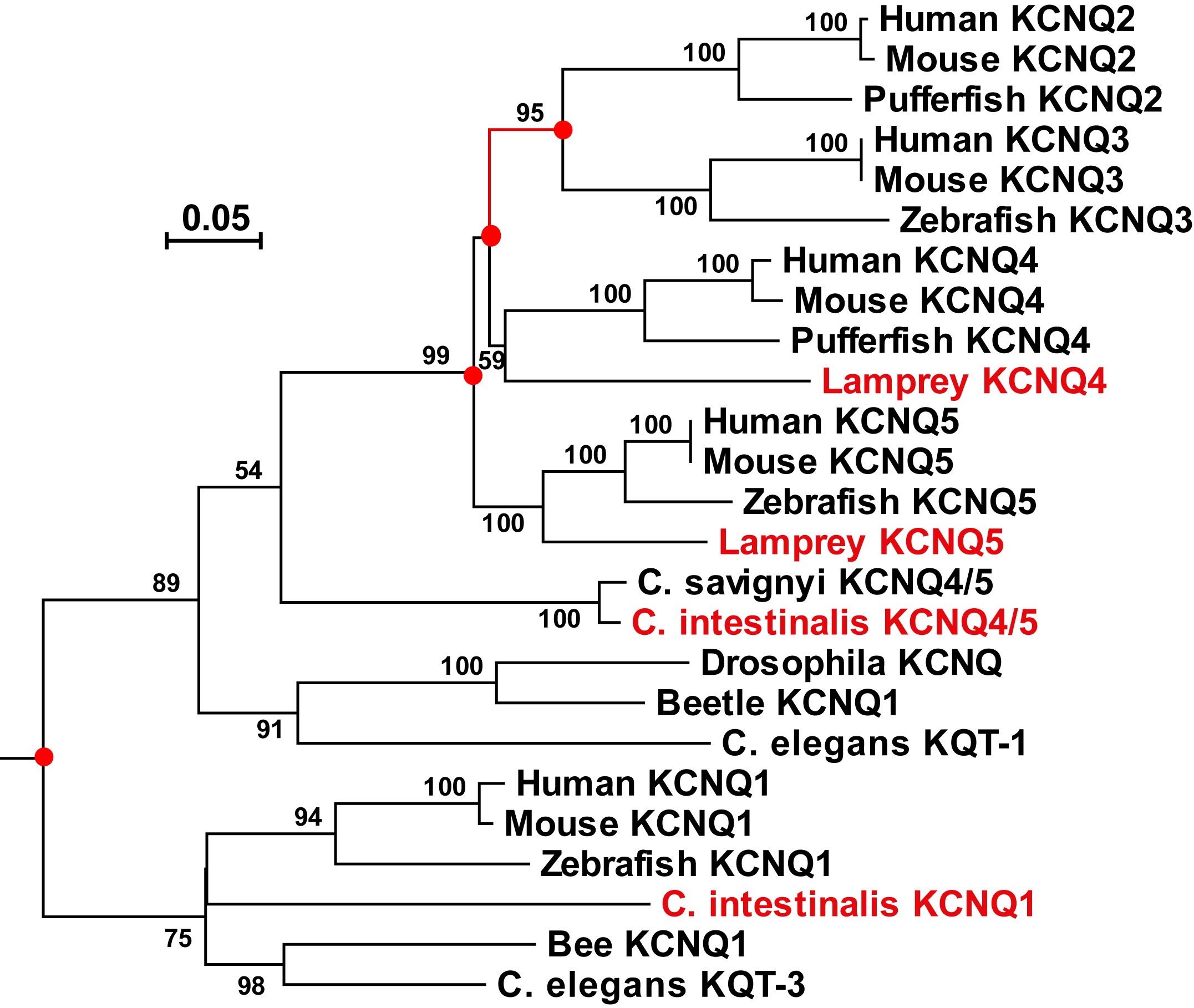

Supplement: Figure S6 — KCNQ gene family phylogeny (minimal evolution) based on analysis of exons 4–14. Nodes are labeled by bootstrap values, scale indicates changes per residue. The branch on the phylogram in which the anchor motif first evolved is shown in red. Nodes associated with gene duplications are indicated by red dots. Results are similar to those derived from analysis of conserved exons 5–7 only (shown in Figure 2C). C. intestinalis and P. marinus KCNQ genes cloned here (red text) appear orthologous to KCNQ1, KCNQ5, and (P. marinus) KCNQ4. (0.26 MB TIF) [file pgen.1000317.s006.tif]

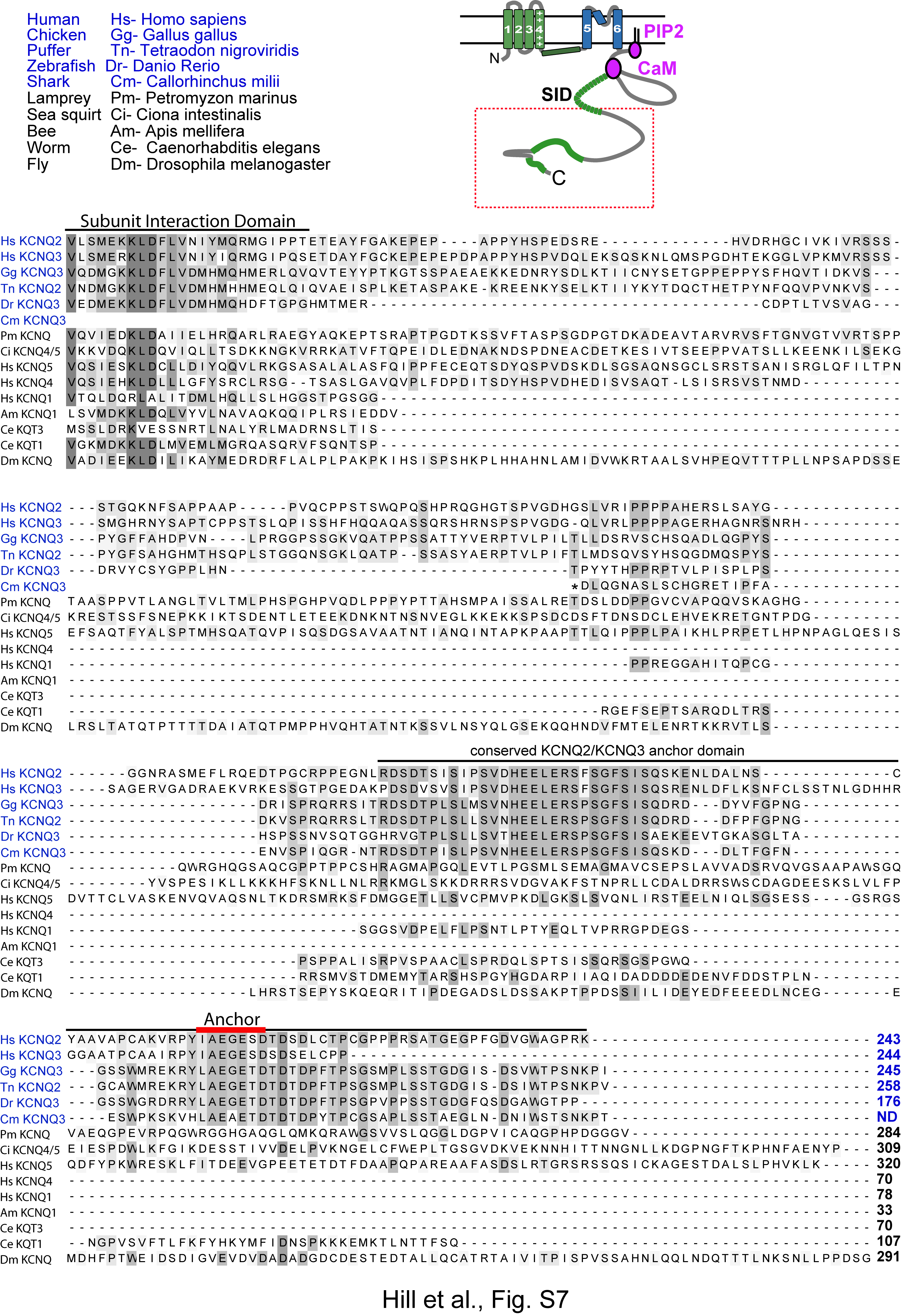

Supplement: Figure S7 — KCNQ exons encoding the C-terminal region begin with conserved sequence encoding the subunit interaction domain, but are otherwise poorly conserved in length and sequence except for the domains of KCNQ2 and KCNQ3 bearing the anchor motif. Aligned peptide sequences corresponding to the entire 3′ coding exons of 15 representative vertebrate and invertebrate KCNQ genes are shown. Except for the initial ∼15 residues (forming part of the subunit interaction domain), only the distal domains containing anchor motifs, which are exclusive to jawed vertebrate KCNQ2 and KCNQ3 sequences (blue lettered subunits), are conserved. Codon lengths for the exons are given at bottom right; the 5′ portion of sequence for shark is unknown. (2.67 MB TIF) [file pgen.1000317.s007.tif]
